# The Application of Robotics in Cardiac Rehabilitation: A Systematic Review

**DOI:** 10.3390/medicina60071161

**Published:** 2024-07-18

**Authors:** Aseel Aburub, Mohammad Z. Darabseh, Rahaf Badran, Ala’a M. Shurrab, Anwaar Amro, Hans Degens

**Affiliations:** 1 Department of Physiotherapy, Applied Science Private University, Amman 11931, Jordana_amro@asu.edu.jo (A.A.); 2 Department of Physiotherapy, School of Rehabilitation Sciences, The University of Jordan, Amman 11942, Jordan; 3 Department of Physiotherapy, Faculty of Applied Medical Sciences, Middle East University, Amman 11831, Jordan; 4 Department of Basic Medical Science, Faculty of Medicine, Al-Balqa Applied University, Al Salt 19117, Jordan; alaa.shurrab@bau.edu.jo; 5 Department of Life Sciences, Institute of Sport, Manchester Metropolitan University, Manchester M1 5GD, UK; h.degens@mmu.ac.uk; 6 Institute of Sport Science and Innovations, Lithuanian Sports University, LT 221 Kaunas, Lithuania

**Keywords:** cardiac rehabilitation, robotics, performance, exercise capacity, physiotherapy

## Abstract

*Background and Objectives*: Robotics is commonly used in the rehabilitation of neuro-musculoskeletal injuries and diseases. While in these conditions, robotics has clear benefits, it is unknown whether robotics will also enhance the outcome of cardiac rehabilitation. This systematic review evaluates the use of robotics in cardiac rehabilitation. *Methods*: A systematic literature search was conducted using PubMed (MEDLINE), CINAHL, AMED, SPORTDiscus, and the Physiotherapy Evidence Database. Longitudinal interventional studies were included if they met specified criteria. Two reviewers independently conducted title, abstract, and full-text screening and data extraction. The quality assessment and risk of bias were conducted according to the PEDRO scale and Cochrane Risk of Bias tool 2, respectively. *Results*: Four trials were included in this review out of 60 screened studies. The quality of the included studies was good with a low risk of bias. The trials used different robotic systems: Lokomat^®^ system, Motomed Letto/Thera Trainer tigo, BEAR, and Myosuit. It was found that interventions that included the use of robotic assistance technologies improved the exercise capacity, VO_2_ max/peak, left ventricular ejection fraction, QOL, and physical functioning in people with cardiac diseases. *Conclusions*: Robotic assistance technologies can be used in cardiac rehabilitation programs. Further studies are needed to confirm the results and determine whether the use of robotics enhances intervention outcomes above standard interventions.

## 1. Introduction

Cardiovascular diseases (CVDs) remain the leading cause of mortality globally, with an estimated 17.9 million deaths each year, accounting for 31% of all global deaths [1]. This alarming statistic underscores the urgent need for effective rehabilitation strategies to manage and mitigate the long-term impact of cardiac events. In recent years, the healthcare field has become interested in developing approaches for management and rehabilitation that are easier and more convenient for patients [2]. Digital health has emerged as a cornerstone to enhance healthcare delivery [2]. It has been shown that technologies, such as smartwatches, mobile applications, and remote monitoring devices, successfully enhanced patient engagement, resulting in optimized treatment outcomes and facilitated proactive management in chronic diseases [3,4,5]. In cardiac rehabilitation, these technologies play a pivotal role in the continuous monitoring of vital signs and facilitating remote communication between healthcare providers and patients (“telerehabilitation”) that help to deliver tailored and customized rehabilitation programs whether at home, in the community, or in the clinic [6,7]. Digital health is not only about using advanced technologies to assess and monitor the condition of the patient but also about leveraging data analysis that enables healthcare professionals to deliver timely interventions, thus improving the efficacy and effectiveness of rehabilitation [8]. Moreover, digital health technologies empower patients to actively participate in their own care, thereby improving their sense of autonomy and accountability and promoting adherence to prescribed rehabilitation regimens [9,10]. An emerging scope of focus among health technologies is the use of robotics in cardiac rehabilitation [11].

Robotics, with its precision and adaptability, offers a promising avenue for enhancing cardiac rehabilitation outcomes by providing tailored and intensive therapy that can adapt to the specific needs of each patient [11]. For instance, robotic-assisted rehabilitation devices enable precise and targeted therapeutic interventions, allowing for control over the movement of affected limbs and supporting the action of weakened muscles, thereby improving mobility [11]. Additionally, robotics provides interactive feedback and adaptive assistance to facilitate the progression of rehabilitation protocols and combined with digital technologies provide close monitoring [12]. Moreover, robotics can be integrated with virtual reality and digital games, adding a fun or gaming element to rehabilitation exercises, thereby enhancing patient engagement, adherence, and motivation [13,14]. Ultimately, the beauty of adding robotics to rehabilitation, especially cardiac rehabilitation, may not only improve the quality of care but also reduce the long-term consequences of cardiovascular diseases on patients and the healthcare system [15].

Robotics is already highly advanced and commonly used in neurological and musculoskeletal physiotherapy, such as in the rehabilitation after stroke [16], spinal cord injuries [17], orthopedic and sports injuries [18], and in the rehabilitation of Parkinson’s disease [19]. Many studies confirm the effectiveness in these conditions and the improvement in rehabilitation outcomes. However, the benefits of robotics in cardiac rehabilitation are not much studied. Therefore, the objective of this systematic review is to summarize and discuss the application of robotics in interventions in cardiac rehabilitation.

The primary research question of this review is as follows: what is the current information about the application of robotics in cardiac rehabilitation? The hypothesis is that robotic-assisted rehabilitation improves cardiorespiratory fitness, functional mobility, and patient adherence in people with CVDs.

## 2. Methodology

### 2.1. Objective

The objective of this study was to review and discuss the reported effects of using robots during interventions on cardiac rehabilitation outcomes in people with heart failure.

### 2.2. Design

A systematic review with a quality assessment and narrative synthesis of the relevant published literature was conducted. This systematic review follows the PRISMA (Preferred Reporting Items for Systematic Reviews and Meta-Analyses) guidelines to ensure comprehensive and transparent reporting [20].

### 2.3. Study Protocol

The systematic review protocol is registered in the international prospective register of systematic reviews database (PROSPERO) (PROSPERO 2024, CRD42024534712). To not limit the search, outcome measures were not limited to any keywords.

### 2.4. Search Strategy

A search was completed through EBSCO using the following electronic databases: PubMed (MEDLINE), CINAHL, AMED, SPORTDiscus, and Physiotherapy Evidence Database (PEDro). The selected databases were chosen because of the likely availability of robotics-related interventions in cardiac rehabilitation/physiotherapy, physiotherapy, and exercise-related articles. The databases were searched for trials published between 1 January 1970 and 6 May 2024. The results of the searches were managed using Endnote Version 21 (Clarivate Analytics, Philadelphia, PA, USA). The Medical Subject Headings (MeSH) were used to allow for the reproducibility and accuracy of the search. Table 1 summarizes the combinations of keywords included in the search strategies.

To allow for the reproduction of the results, adapted searches for each database were conducted according to the PRISMA guidelines. To get a broader idea of ongoing research, the gray literature was explored using the World Health Organization (WHO) International Clinical Trials Registry platform.

### 2.5. Inclusion and Exclusion Criteria

The PICOS system (Population, Intervention, Comparison, Outcome measures and study design) was used to define the inclusion and exclusion criteria. Articles were included if they were longitudinal interventional studies investigating the effects of using robots in cardiac rehabilitation in adult men and women, aged >18 years old and diagnosed with heart failure. Articles were excluded if they were not longitudinal interventional studies; did not use robotics in cardiac rehabilitation; were not published in English; were conference abstracts; or included participants with other neurological, musculoskeletal, or cognitive disorders. The comparison was not limited to any intervention and included single-arm longitudinal studies where found.

### 2.6. Study Selection

Following the search and subsequent removal of duplicates, titles and abstracts were independently screened by two reviewers (RB and AAm) for relevance. The full texts of relevant trials were then independently screened by the same reviewers for eligibility against the inclusion and exclusion criteria.

### 2.7. Data Extraction

The following data were extracted from the included trials and are presented in a table: author name/s, trial location, study design, sample size, sex, age, type of robot services provided (technology utilized), exercise prescription, outcome measures reported, and key findings. Also, the frequency, time, intensity, and type (FITT) of exercise were noted if they were available.

### 2.8. The Quality Assessment and Risk of Bias of the Included Trials

The quality assessment of the included studies was conducted using the PEDro scale if they were randomized control trials (RCTs), which is a valid and reliable tool for assessing the quality of interventional studies specifically related to physiotherapy interventions [21,22]. PEDro scores for the trials were not used as an inclusion or exclusion criterion but as a basis for best-evidence synthesis and to determine the strengths and weaknesses of each trial. PEDro scores range from 0 to 10; scores of 9–10 indicate excellent quality, 6–8 indicate good quality, 4–5 low quality, and scores below 4 indicate poor quality [23].

The risk of bias of the included RCTs was assessed using the Cochrane Risk of Bias tool 2 (CROB 2). Two reviewers (RB and AAm) assessed the risk of bias independently. The following were assessed using the CROB 2: (1) bias arising from the randomization criteria; (2) bias due to deviations from intended interventions; (3) bias due to missing outcome data; (4) bias in the measurement of the outcome; and (5) bias in the selection of the reported results.

## 3. Results

### 3.1. Study Characteristics

**Figure 1:**
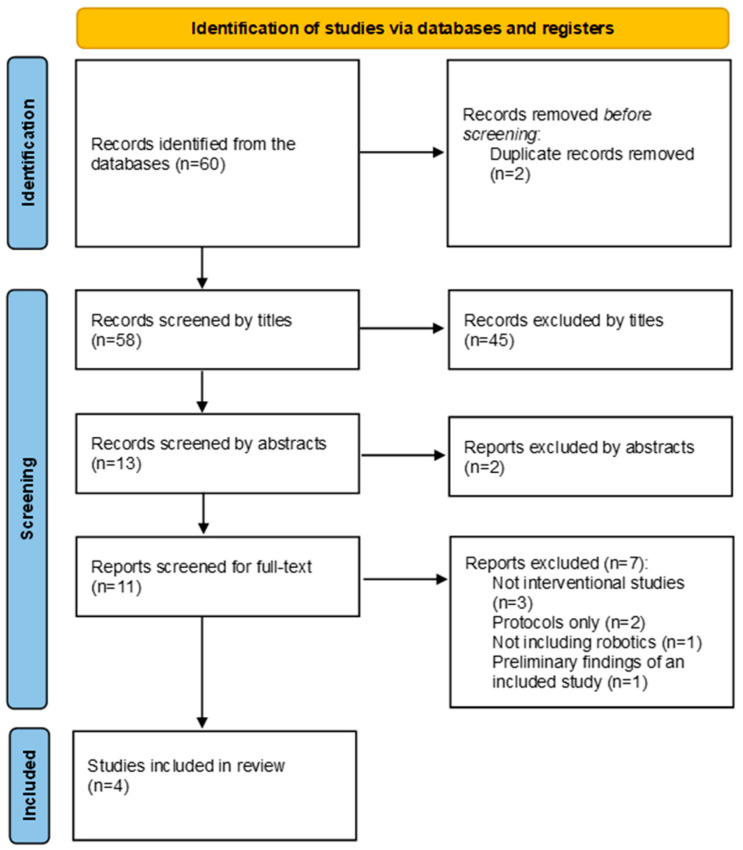
A PRISMA flowchart of the records.

The systematic search identified 60 citations from the databases, of which 2 citations were duplicates. Consequently, 58 citations were screened from titles. A total of 45 of the 58 citations were considered not to be relevant. Of the 13 remaining studies, 2 articles were excluded after reading the abstracts, and a total of 11 studies were screened by reading the full text. Out of the 11 studies, 7 studies were excluded due to the following reasons: 3 studies were not interventional studies, 2 were protocols of trials only, 1 study was a preliminary study of an included study, and 1 study did not include robotics as an intervention. Consequently, four trials were included in the review. Figure 1 represents the PRISMA flowchart for the search records. A meta-analysis was not feasible because of the heterogeneity of the included trials.

### 3.2. Quality Assessment

**Figure 2:**
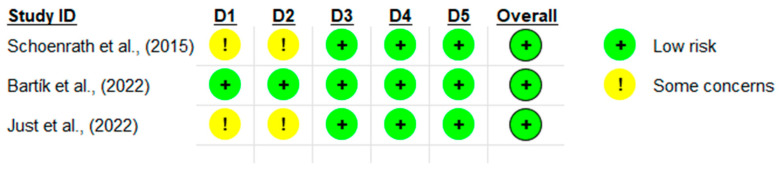
The results of the quality assessment of the controlled trials (n = 3) using the CROB2 [25,26,27].

The quality of the trials was independently assessed by RB and AAm using the PEDro score and CROB2. Figure 2 presents a summary of the CROB2 results. Table 2 is the data extraction table for the three included trials. The fourth study Hashimoto, et al. (2022) [24] was a single-arm longitudinal study. Therefore, it was not possible to run a quality assessment for it through PEDro or the CROB2 tool. The PEDro scores ranged from 6 to 8 (Table 3). Three trials had a good quality rating according to the criteria established by the NIH: NHLBI Quality Assessment Tool.

### 3.3. Robotics Used

Various robotic systems, including the Lokomat^®^ system, Motomed Letto/Thera Trainer tigo, BEAR, and Myosuit, were utilized in the four trials. The Lokomat^®^ system was used in one trial [25]. It is a treadmill with a bilateral powered gait orthosis that is fixed to the rigid frame of the bodyweight support system, and it contains straps across the waist, thighs, and shanks to secure the orthosis to the patient. The system can be manually modified by changing the offsets and amplitudes of the hip and knee joint trajectories to suit each patient. During training, the speed of the treadmill can be modified with the assistance of augmented feedback.

Motomed letto/Thera Trainer tigo was used in one trial [26]. Motomed letto is a motor-assisted bed model training device that is frequently used for bedridden patients. THERA-Trainer tigo is a motor-assisted training device with an automatic system for leg or arm mobilization and training in different positions. Both devices allow for passive, active-assisted, active, or active-against-resistance movements. The device is safe and can be used without the supervision of a physiotherapist. The positions and level of resistance can be modified according to the patient’s condition.

The BEAR was used in one trial [24]. It is a Balance Exercise Assist Robot that has two motor-controlled wheels by an inverted pendulum system and a foot plate on the sides. Changes in the operator’s center of gravity allow the BEAR to move left, right, forward, and backward. The system allows the user to play three games that target distinct skill sets that can help to create a balanced workout by encouraging movements in four directions. The users can automatically execute repeated movements, and the difficulty level of the exercises can be modified to suit each individual.

Myosuit was used in one trial [27]. It is a soft, wearable, exoskeleton-type robot that assists in the coordinated extension of the hip and knee joints throughout a variety of daily activities, including standing, walking, sitting, and climbing stairs.

### 3.4. Intervention Prescription: FITT

Each of the four trials had a different exercise prescription. Schoenrath et al. (2015) [25] asked interventional group (IG) participants to train three times a week for four weeks. The target exercise intensity was 11–12 RPE. During training, two to three exercise sets of 6–10 quadricep resistance exercises were performed for both legs against the resistance of the Lokomat gait orthosis for 10–20 min during the first session and up to 30 min in the last session. The control group (CG) received standard physiotherapy care for 5 days a week for four weeks including the training of trunk stability and walking without exertion. All training sessions lasted 10–30 min. Both groups received standard respiratory training.

In Bartík et al. (2022) [26], the IG performed active-assisted and active repetitive movements of the upper and lower limb exercise in different positions (on the bed, sitting, standing), moving to a standing position, short walk, short walk up and down the stairs, and 20 min of robot-assisted training with repetitive movements. The CG performed active-assisted and active repetitive movements of the upper and lower limb in different positions (on the bed, sitting, standing), moving to standing position, short walk, and short walk up and down the stairs for 45 min. Both groups received an early intensive physiotherapy program.

Hashimoto et al. (2022) [24] assessed participants (one group) at baseline and after 4 months. Participants were asked to perform balance and aerobic exercises while using the BEAR. The training was once a week for four months for 20 min. The load was increased gradually according to the subjective exercise intensity.

In Just et al. (2022) [27], a cross-over intervention with and without Myo suit robot was conducted. The participants performed ADL (single session of timed walking for 6 min, standing, sitting down on a chair, standing up from a chair, and climbing stairs) with or without the Myosuit. For standing, a static mode of the Myosuit was used. Throughout the study period, the participants received standardized rehabilitation exercises including dynamic walking training combined with a resistance exercise of the upper body and dynamic and static balance training for 60 min.

### 3.5. Effects of Robotics on Peak Oxygen Consumption (VO_2_ Peak) and Ejection Fraction (EF)

Only one trial used VO_2_ peak as an outcome measure [24]. Hashimoto et al. (2022) reported no significant differences in VO_2_ peak from baseline to after the four-month intervention (*p* = 0.222) [24]. Schoenrath et al. (2015) [25] reported that left ventricle EF was significantly higher in the robot assist group compared with the conventional group after the intervention period (*p* = 0.04).

### 3.6. Effects of Robotic Interventions on Exercise Capacity

Three trials investigated the impact of using robotics in cardiac rehabilitation on exercise capacity [25,26,27]. Although both the CG and IG had an improved peak quadricep force (IG right, *p* = 0.011, left *p* = 0.005; CG, *p* < 0.001), the increase was larger in the IG (range: 3% to 80%) [25].

Additionally, Schoenrath et al. (2015) reported that the improved walking capacity (IG *p* = 0.005, CG *p* < 0.001) and quadricep peak force (IG right, *p* = 0.011, left *p* = 0.005; CG, *p* < 0.001) after one month of follow up were similar in the CG and IG [25].

Hashimoto et al. (2022) [24] reported an improved gait speed (*p* < 0.001), timed up-and-go (TUG) (*p* = 0.004), and knee extension (*p* = 0.042) from baseline to after 4 months of follow up. Furthermore, Just et al. (2022) [27] reported that there was an improvement in favor of patients with robotic assistance compared with the CG [27].

### 3.7. Effects of Robotic Interventions on Quality of Life (QOL) and Physical Functioning

Three trials investigated the effects of using robotic interventions on the QOL and physical functioning [24,26,27]. Bartík et al. (2022) [26] reported that the improvements were larger in the EG in the FIM-AD indicator and FIM-MOTOR indicator compared with the control group (both *p* < 0.01) [26]. Also, the differences were larger in the EG in the motor and ADL scores compared with the control group after the intervention (*p* < 0.00) [26].

Hashimoto et al. (2022) [24] reported an improvement in the Short Physical Performance Battery (SPPB) score (*p* = 0.004) but no improvement in the Falls Efficacy Scale International (FES-I) score (*p* = 0.139) from baseline to after four months of follow up [24].

Just et al. (2022) [27] reported that neither group had a significant improvement in the rates of perceived exertion and dyspnea (RPE: *p* = 0.932 and RPD; *p* = 0.141) [27].

### 3.8. Safety of Robotic Interventions (Adverse Events)

No adverse events were reported in the four trials.

## 4. Discussion

The aim of this systematic review was to explore the application of robotic systems in cardiac rehabilitation. The four included trials used various robotic systems: the Lokomat^®^ system, Motomed Letto/Thera Trainer tigo, BEAR, and Myosuit. The primary outcomes assessed were exercise capacity, VO_2_ max/peak, QOL, and physical functioning. The novelty of this systematic review lies in its comprehensive analysis of diverse robotic systems utilized in cardiac rehabilitation, providing a broad perspective on their efficacy. This review highlights the emerging role of robotic assistance in enhancing rehabilitation outcomes, an area that has seen limited systematic exploration. It was found that robotic assistance technologies are a potentially useful addition to cardiac rehabilitation programs.

Three trials [24,25,27] indicated significant improvements in exercise capacity, including enhancements in peak work rate, gait speed, and quadricep force. These findings suggest that robotic-assisted rehabilitation can positively impact exercise capacity, although its effect on VO_2_ may vary depending on individual and protocol used differences.

The impact of interventions that included robotics on the quality of life and physical functioning was assessed in three trials [24,26,27]. Significant improvements were noted in measures such as the SF-36 and Functional Independence Measure (FIM) scores. Specific indicators like physical function, gait speed, and the Short Physical Performance Battery (SPPB) score showed marked enhancement. However, some measures, such as the FES-I and perceived exertion rates, did not show significant improvements with robotic assistance.

Though the use of robotics showed no improvement in FES-1 and RPE, this could be due to some individual differences and techniques applied in the trials. It should be noted, however, that the interventions in the control and robotic group were not identical in Schoenrath, and there was no control group without robotics in the study by Hashimoto et al. It is therefore difficult to disentangle the additional benefits of robotics on the outcome of cardiac rehabilitation. Where the only difference was the application of robotics between the control and experimental group, there was either no additional benefit of robotics (Just et al., 2022) [27] or an enhanced improvement in FIM and ADL (Bartik et al., 2022) [26].

The application of robotics in cardiac rehabilitation may be significant due to its potential to enhance patient outcomes through tailored, precise, and consistent training regimens. Robotics can facilitate higher adherence to rehabilitation programs by making exercises more engaging and accessible, even for patients with severe mobility limitations. This field is rapidly growing, driven by technological advancements and an increasing recognition of the benefits of personalized rehabilitation strategies. As the technology evolves, it is likely to become a standard component of comprehensive cardiac rehabilitation programs in the future.

This systematic review has several limitations. The small number of trials included and their varying designs and methodologies limit the generalizability of the findings. Differences in intervention protocols, duration, and intensity, the lack of a non-robotic group, or differences between the intervention with and without robotics make it challenging to draw definitive conclusions about the overall efficacy of robotic-assisted rehabilitation. Additionally, the heterogeneity of the patient populations and the short duration of some trials may not fully capture long-term benefits and adherence. Furthermore, the heterogeneity of the data, outcome measures used, and exercise prescription limit the ability to perform a meta-analysis. Future research, specifically randomized control trials, with larger sample sizes, standardized protocols, and appropriate control groups is needed to better understand the potential benefits of robotics in cardiac rehabilitation.

In conclusion, this systematic review indicates that robotic-assisted rehabilitation might be used to enhance exercise capacity and certain aspects of physical functioning and quality of life in cardiac patients. While the effects on VO_2_ remain inconclusive, the overall benefits of robotics in rehabilitation are promising. The integration of robotic systems into cardiac rehabilitation programs represents a valuable advancement in patient care, offering a means to improve outcomes through innovative and personalized interventions. Further research is essential to verify any benefits and expand the evidence base for robotic-assisted cardiac rehabilitation.

## Figures and Tables

**Table 1:** Summary of keywords used and search strategy.

Search Strategy	Search Strategy
P (population)	The keywords to be used are the following: S1 = “cardiovascular diseases” OR “CVD” OR “cardiac diseases” OR “coronary heart diseases” OR “cardiac surgeries” OR “heart diseases” OR “cardiac disorders’’ OR “heart disorders” OR “heart failure” OR “myocardial ischemia” OR “MI”
I (Intervention)	S2 = “robotics” OR “robot” OR “robots” OR “socially assistive robotics” OR “social robots” AND “physiotherapy” OR “physiotherapist” OR “rehabilitation” OR “cardiac rehabilitation” OR “cardiovascular rehabilitation” OR “cardiac rehab” OR “cardiovascular rehab” OR “cardiac physiotherapy” OR “cardiovascular physiotherapy”
C (comparison)	The comparison was not limited to any intervention and even included single-arm longitudinal studies where found.
O (outcome measures)	Outcome measures were not limited to any keywords
S (study design)	Randomized controlled trials, controlled clinical trials, or longitudinal interventional trials
Combined final search	S1 AND S2

**Table 2:** A summary of included trials that investigated the usage of robot-related interventions on outcomes in cardiac rehabilitation.

Author (Year)	Trial Location	Study Design	Sample Size	Age Years (Mean ± SD)	Technology Utilized	Physical Capacity	Exercise Prescription	Primary Outcome	Secondary Outcome	Key Findings
Schoenrath et al., (2015) [25]	Switzerland	RCT	Robot-assist Group: 10 participants Conventional Group: 20 participants	60.3 ± 11.2	Lokomat^®^ system	- 6MWT	Duration of treatment and follow up: 1 month. Both groups received standard respiratory training Robot-assist Group: F: 3×/week. I: RPE scale (6–20); target exertion 11–12 T: Two to three exercise sets of 6–10 quadricep resistance exercises for both lower extremities. T: 40 min/session Conventional Group F: 5×/week. T: Training trunk stability and walking T: 10–30 min/session.	- 6MWT	- QPF	- Both groups improved 6MWT (IG *p* = 0.005, CG *p* < 0.001). - Both groups improved QPF (IG right, *p* = 0.011, left *p* = 0.005; CG, *p* < 0.001) - Differences in walking capacity larger in the Robot-assist than conventional group (*p* < 0.01) - Left ventricle EF was higher in the IG compared with CG (*p* = 0.04). - Median % change comparable (left *p* = 0.97, right *p* = 0.61) - No adverse events or cardiopulmonary emergencies.
Bartík et al., (2022) [26]	Czech Republic	RCT	Experimental group: 46 participants Control group: 46 participants F:M = 42:50	60.9 ± 2.32	Motomed letto/Thera Trainer tigo	- HR/rest - HR/effort	Duration of treatment and follow up: 14 days (28 sessions) Both groups received an early intensive physiotherapy program F: 7×/week (2×/day) I: depended on patient’s condition T: Active assistant ROM exercises in different positions (standing, sitting, lying in a supine position) T: 45 min/session. Intervention group (IG) T: active-assisted and active repetitive analytical movements of the upper and lower limbs exercise in different positions (on the bed, sitting, standing). Mobilization, short walk, short walk up and down the stairs, and robot-assisted training with repetitive movements T: 45 min/session Control group (CG) T: active-assisted and active repetitive analytical movements of upper and lower limb exercise on the bed, sitting, standing. Mobilization, short walk, short walk up and down the stairs T: 45 min/session	- FIM		- After 14 days follow up, both groups had an improved FIM score (*p* < 0.05). - The differences were larger in the IG in FIM-AD indicator and FIM-MOTOR indicator compared with the CG (both *p* < 0.01) - The differences were larger in the IG in the Motor and ADL scores compared with CG after the intervention (*p* < 0.00) - No significant differences were reported between groups in FIM-SOCIAL indicator (*p* = 0.35) - No adverse event was reported
Hashimoto et al., (2022) [24]	Japan	A single arm longitudinal study	52 participants (one group) F:M = 24:28 Assessed at baseline and after 4 months	76.9 ± 6.8	The BEAR	VO_2_ peak	Duration of intervention and follow up: 4 months (16 session) F: 1×/week I: Depended on patient condition T: balance and aerobic exercise T: 21 min/session	- 10 m gait speed - SPPB - TUG - Muscle strength of knee extension		- From baseline to 4 months follow up improvements in gait speed (*p* < 0.001), SPPB score (*p* = 0.004), TUG time (*p* = 0.004), and knee extension (*p* = 0.042). - No adverse events occurred
Just et al., (2022) [27]	Switzerland	Cross-over trial	Standard group (G1): 10 participants. REU (G2): 10 participants. F:M = 4:16	49.4 ± 11.0	The Myosuit	Vital signs	- All patients performed mobilization protocols in a cross-over design both with and without the Myosuit G1 T: Performed a single session of timed walking for 6 min, standing, sitting down and standing up from a chair, and climbing stairs. For standing, a static mode of the Myosuit G2 T: Single standardized REU with dynamic walking, resistance exercise of upper body, and dynamic and static balance training T: 60 min/session	- RPE - RPD	- Individual acceptability	- There were no significant differences in the total walking distance of the patients without and with robotic assistance (*p* = 0.241). - There was no significant difference in RPE (*p* = 0.932)) or RPD (*p* = 0.141) with or without robotic assistance. - A total of 85% were interested in participating in robot-assisted training on a regular basis. - No adverse events occurred

VO_2_ peak: peak oxygen consumption; RPE: Borg Rating of Perceived Exertion; EF: ejection fraction; 6MWT: 6-Minute Walk Test; QPF: Quadriceps peak force; ROM: Range of Motion; FIM: Functional Independence Measure; HR: Heart Rate; BEAR: Balance Exercise Assist Robot; SPPB: Short Physical Performance Battery; TUG: timed up-and-go; RPE, RPD: rates of perceived exertion and dyspnea; REU; Rehabilitation Exercise Unit; ADL: Activity of Daily Living.

**Table 3:** The results of the PEDro scale for quality assessment for the included randomized controlled trials.

Author (Year)	1. Eligibility Criteria Were Specified	2. Subjects Were Randomly Allocated to Groups	3. Allocation Was Concealed	4. The Groups Were Similar at Baseline Regarding Prognostic Indicators	5. There Was Blinding of All Subjects	6. There Was Blinding of All Therapists Who Administered the Therapy	7. There Was Blinding of All Assessors Who Measured at Least One Key Outcome	8. Measures of at Least One Key Outcome Were Obtained from More Than 85% of the Subjects	9. All Subjects for Whom Outcome Measures Were Available Received the Treatment or Control Condition as Allocated	10. The Results of between-Group Statistical Comparisons Are Reported for at Least One Key Outcome	11. Point Measures and Measures of Variability for at Least One Key Outcome Were Reported	Total PEDro Score
Schoenrath et al., (2015) [25]	1	0	0	1	0	0	0	1	1	1	1	6
Bartík et al., (2022) [26]	1	0	0	1	1	1	0	1	1	1	1	8
Just et al., (2022) [27]	1	0	0	1	0	0	0	1	1	1	1	6

## Data Availability

The data that support the findings of this study are available within the manuscript.
